# Codon Size Reduction as the Origin of the Triplet Genetic Code

**DOI:** 10.1371/journal.pone.0005708

**Published:** 2009-05-27

**Authors:** Pavel V. Baranov, Maxime Venin, Gregory Provan

**Affiliations:** 1 Biochemistry Department, University College Cork, Cork, Ireland; 2 Computer Science Department, University College Cork, Cork, Ireland; University of Otago, New Zealand

## Abstract

The genetic code appears to be optimized in its robustness to missense errors and frameshift errors. In addition, the genetic code is near-optimal in terms of its ability to carry information in addition to the sequences of encoded proteins. As evolution has no foresight, optimality of the modern genetic code suggests that it evolved from less optimal code variants. The length of codons in the genetic code is also optimal, as three is the minimal nucleotide combination that can encode the twenty standard amino acids. The apparent impossibility of transitions between codon sizes in a discontinuous manner during evolution has resulted in an unbending view that the genetic code was always triplet. Yet, recent experimental evidence on quadruplet decoding, as well as the discovery of organisms with ambiguous and dual decoding, suggest that the possibility of the evolution of triplet decoding from living systems with non-triplet decoding merits reconsideration and further exploration. To explore this possibility we designed a mathematical model of the evolution of primitive digital coding systems which can decode nucleotide sequences into protein sequences. These coding systems can evolve their nucleotide sequences via genetic events of Darwinian evolution, such as point-mutations. The replication rates of such coding systems depend on the accuracy of the generated protein sequences. Computer simulations based on our model show that decoding systems with codons of length greater than three spontaneously evolve into predominantly triplet decoding systems. Our findings suggest a plausible scenario for the evolution of the triplet genetic code in a continuous manner. This scenario suggests an explanation of how protein synthesis could be accomplished by means of long RNA-RNA interactions prior to the emergence of the complex decoding machinery, such as the ribosome, that is required for stabilization and discrimination of otherwise weak triplet codon-anticodon interactions.

## Introduction

The ribosome is a sophisticated multifunctional nano-machine that is responsible for protein biosynthesis in all cellular organisms. Among all functions fulfilled by the ribosome, the most striking is its ability to decode mRNA by accurately discriminating codon/anticodon triplet interactions occurring between mRNAs and cognate tRNAs from those occurring with non- or near-cognate tRNAs. Due to significant progress in investigation of the three-dimensional structure of the ribosome and its complexes, we now have a better understanding of how such discrimination is accomplished [1]–[5]. Codon/anticodon interactions are very weak and even the formation of such interactions cannot be observed without the ribosome. Therefore the triplet character of the genetic code is mechanistically dependent on the ribosome. A major component of the ribosome is RNA and it has been argued that the ribosome is a ribozyme [6]–[8], as certain catalytic centers of the ribosome, such as the peptidyl transferase centre, are formed purely by RNA components [9], [10]. However, it is clear that the efficiency and accuracy of mRNA decoding relies on protein components of the ribosome as well as on translation factors which are also protein molecules. The mutual dependence of protein biosynthesis and the decoding apparatus is frequently regarded as a ‘chicken and egg’ problem. The virtue of finding a solution for this problem has been proclaimed a number of times, and many ideas that tackle this problem have been proposed. The major themes currently discussed in the literature in relation to the code origin can be divided into: (i) accidental origin of the code, (ii) optimization of the code's effectiveness, (iii) stereo chemical relationships between amino acids [11]–[20], and (iv) dynamic self-organization [21], [22]. A complete representation of these and other ideas is outside the scope of the current manuscript, as only a few of them concern the origin of the triplet size of codons specifically. Those that do address the evolution of codon size consider the evolution of triplet codons from doublet codons [23]–[25]. However, the discriminative capacity of doublet codons is even more problematic in the absence of a sophisticated decoding apparatus (as it requires accuracy higher than of modern ribosome) and therefore, such a decoding system is unlikely to evolve in a pure RNA World. In contrast, long complementary interactions between RNA molecules are energetically stable in solution, and may be discriminated without trans-factor assistance (see Discussion section for detail). This article is dedicated to the analysis of one particular scenario of triplet codon evolution, where we consider its emergence from decoding systems with longer-than-triplet decoding.

While the accidental nature of the genetic code is still a matter of scientific dispute [16], [26]–[28], it is clear that the genetic code is not frozen and can be changed, as evident from its multiple variants [29]–[33]. Although indirect, a very strong argument supporting the evolutionary nature of the genetic code is its optimality. The regularity of the genetic code was noted immediately after its decipherment [34]–[36]. The genetic code is remarkably resistant to substitutional point mutations, and to missense translational errors resulting from improper discrimination of similar codons. Indeed, the genetic code has been proclaimed to be “one in a million”, as measured in terms of its robustness to such errors [37]. Recent analyses of its properties have revealed that the genetic code is even more remarkable than that [38]. For instance, the genetic code is near-optimal in terms of its resistance to frameshift errors [39] and also in its ability to code for secondary information in addition to protein sequences [40]. Evolution has no foresight; when we see an optimal solution in Biology, we always find traces of its evolution from less optimal variants. By applying this logic, it is easy to argue that the current genetic code is a product of evolution rather than a very improbable accident [19], [28]. Interestingly, the triplet character of the genetic code is also an optimal solution in terms of its information compactness, as three is the minimal combination of nucleotides required for encoding 20 standard amino acids. Could it be that the triplet character of the genetic code is also a product of evolution from less optimal variants?

Perhaps the most important experimental evidence for a scenario in which triplet decoding is considered to be an evolutionary product of longer-than-triplet decoding is the fact that the modern translational apparatus supports such decoding. Quadruplet decoding was discovered as early as in the 1970s as a mechanism for a frameshift suppression [41]. It has been possible to isolate tRNA molecules with extended anticodon loops, whose incorporation into the ribosome results in quadruplet decoding that suppress +1 frameshift mutations (single nucleotide insertions in mRNA) [42]–[44]. The ability of certain mutant tRNA molecules to support quadruplet and even quintuplet decoding is now well documented [45]–[49]. For a comprehensive review on the topic of frameshift suppression by tRNA mutants see the recent Atkins and Bjork review [50]. Nowadays, quadruplet decoding is extensively used to artificially modify the genetic code, in order to incorporate non-natural amino acids into proteins for a variety of medical and biotechnological purposes [46], [51]–[53].

However, the major impediment for an evolutionary scenario in which quadruplet decoding preceded triplet decoding is the problem of transition between codon lengths. Even the length of a particular codon cannot be changed in all locations of an entire genome without violating the Continuity Principle. Indeed a mutation leading to the emergence of a tRNA molecule that is capable of decoding particular codons as non-triplet should produce enormous numbers of aberrant proteins due to frameshift disruptions in open reading frames coding for proteins. Recent findings relating to the versatility of decoding rules in different species demonstrate that this problem may not be as insurmountable as it seems from a first glance. There are several examples of modern organisms that cope well with non-triplet and non-uniform decoding. The ciliate *Euplotes crassus* contains a large number of genes (up to 10%) that require non-triplet decoding for their expression [54], [55]. *Canida ablicans* tolerates ambiguous decoding of CUG codons as both serine and leucine, resulting in a proteome with a statistical distribution of these two amino acids in locations corresponding to CUG codons in mRNAs [56], [57]. An endosymbiont *Blochmannia pennsylvanicus* contains a very large number of long poly-A stretches in its genome, resulting in non-templated insertions and deletions of As in the corresponding RNA transcripts that break (or restore) the translational phase of numerous coding regions [58]. Finally, a recent discovery demonstrated that a particular codon can have dual amino acid translations in a very controllable manner in a single location, through the action of a distant RNA structure [59]. Altogether, the emerging picture of decoding strategies used by different organisms, besides possibilities to artificially manipulate the genetic code, argue that non-triplet codes or codes with mixed codon sizes are possible. However, without further support, the implications of this possibility in relation to the origin of the genetic code are highly speculative. To test whether the triplet code could originate from longer-than-triplet codes, we have designed a mathematical model that allows evolution of a population of non-triplet decoding digital coding systems. Computer simulations based on this model demonstrate that triplet decoding spontaneously emerges in systems with longer-than-triplet decoding and triplet decoding becomes predominant over time.

## Results

### Brief outline of the model

**Figure 1:**
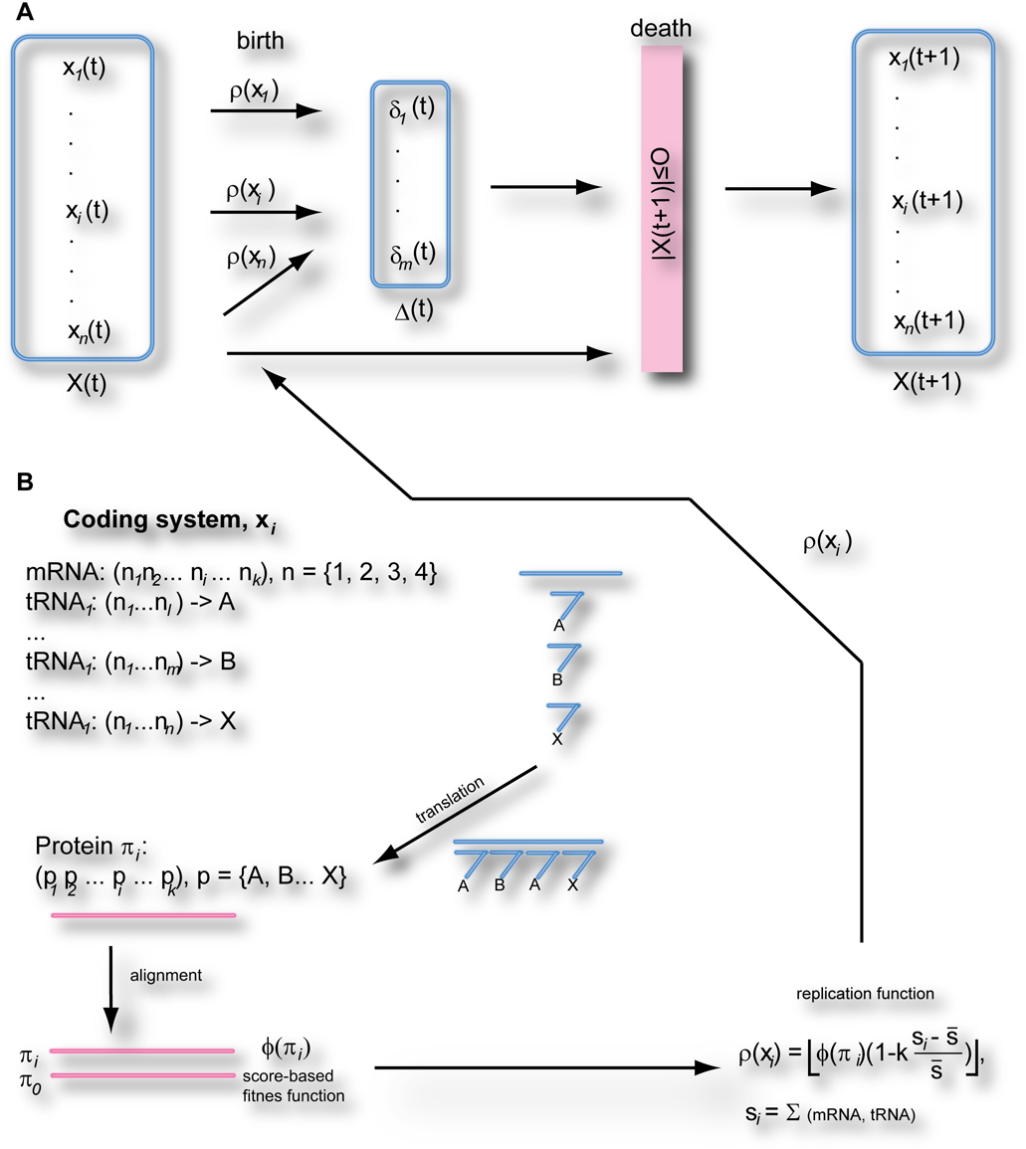
An outline of the Codonevo simulations. A. Population dynamics. During each cycle a population of coding systems undergo two stages, birth (where a number of coding system copies are created according to a replication function ρ) and death (where coding systems are randomly destroyed until their number reaches a certain constant limit O). B. Obtaining the replication function ρ for each coding system. A coding system consists of a single mRNA sequence and a set of tRNA rules that are used to produce a protein sequence. The protein sequence π_i_ is aligned to the reference protein sequence π_0_, and a replication function ρ is calculated based on the score of the alignment and the deviation of the coding system size from the average coding system size in the population. See section Methods for details.

The overall scheme of the model is outlined in Figure 1. In brief, a population consists of a set of digital coding systems X evolving through alternate birth and death cycles. During a birth cycle, a population is increased by a number of coding systems produced according to a replication rate function ρ, which is proportional to the products of (a) the fitness φ of a protein molecule π_i_ produced by coding system χ_i_, and (b) the difference in size of coding system χ_i_ from the average coding system size. System size is the sum of the “nucleotide sequences” (each coding system consists of a single “mRNA” and a set of “tRNA rules” that specifies associations between a combination of nucleotides and a single amino acid). During the death cycle, coding systems are destroyed randomly until their number reaches a certain constant limit. This restriction reflects the limited availability of energy and food resources. For simplicity we consider that the supply of energy and food resources remains constant over time.

The fitness function φ (π_i_) depends on the score of an alignment between a protein molecule π_i_ produced by a coding system χ_i_ and the protein molecule π_0_ produced by an initial coding system at the beginning of a simulation. For simplicity we consider evolution of a protein sequence under purifying selection, i.e., any change in its sequence during the evolution would be deleterious; thus π_0_ remains unchanged throughout the simulation. Protein molecules are produced in each coding system by translating its mRNA (a string of symbols, or ‘nucleotides’, from a four-letter alphabet) by applying translation rules (we use the term “tRNA rule” to distinguish from actual tRNA molecules). Each tRNA rule consists of a codon (a string generated from the four-letter alphabet) and an ‘amino acid’ (a single symbol from a 20-letter alphabet) associated with it. Translation is carried out so that at each step the best-matching tRNA rule is chosen for the current mRNA sequence, and the corresponding amino acid is inserted into the protein molecule sequence. This operation is repeated iteratively for the entire mRNA, where each new tRNA is chosen for the mRNA sequence starting from the end-point of the previous tRNA match. Note that the tRNA rules can match codons of a varied length, unlike during standard translation where the length of codons is uniformly triplet.

Genetic variations introduced during replication of coding systems are of two types. First, point mutations can occur at any position of all nucleotide sequences in a parent coding system, such that substitutions are twice as likely as indels (i.e. insertions or deletions). Second, a copy of a single tRNA rule can be randomly generated in the progeny coding system (gene duplication). See section Methods for a more detailed description of the model as it is implemented in the Codonevo program (available at http://recode.ucc.ie/codonevo).

### Results of simulations

**Figure 2:**
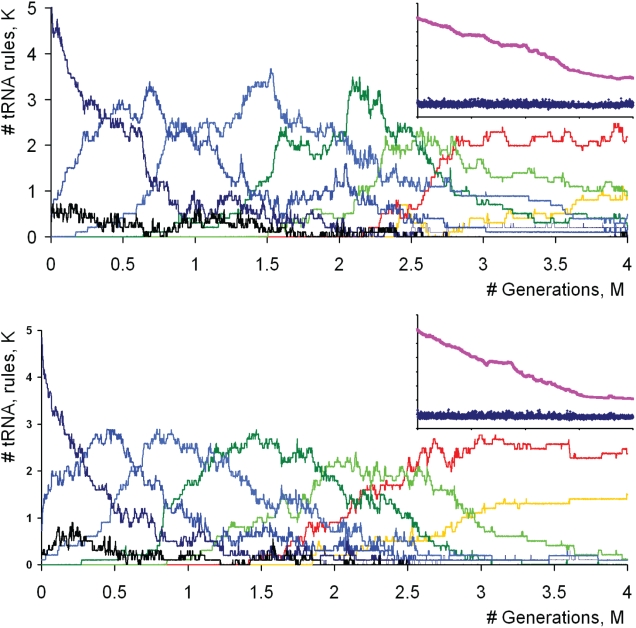
Dynamics of codon sizes in an evolving population of coding systems starting with octuplet genetic codes. Each curve correspond to the number of tRNA rules of a particular size (axis y, factored by thousand, K) in the entire population of the coding systems over time (axis x, number of generations factored my million, M). Curves are differentially colored depending on the codon length as the following: black – 9, dark blue – 8; blue – 7; light blue – 6, dark green – 5, light green – 4, red – 3, yellow – 2, violet – 1. Two simulations with different random starting conditions are shown. Small plots in the top right corners of the main plots show the replication rate of coding systems (dark blue) and the size of mRNA sequence (magenta). Supplementary Movie S1 shows an animated histogram of codon evolution and mRNA length change for the simulation corresponding to the plot at the bottom.

In a number of computer simulations, the initial population of digital coding systems contained a set of tRNA rules with codon sizes of eight (octuplet genetic codes). During these simulations we monitored changes in the size of codons in tRNA rules over time. Figure 2 illustrates the dynamics of codon size changes for two independent simulations (see supplementary material Movie S1 for an animated histogram showing dynamics of codon size evolution). The length of codons in the tRNA rules decreases gradually over time, eventually reaching a state where triplet codons become dominant. Transitional stages when codons of intermediate sizes dominate in the population can also be observed.

**Figure 3:**
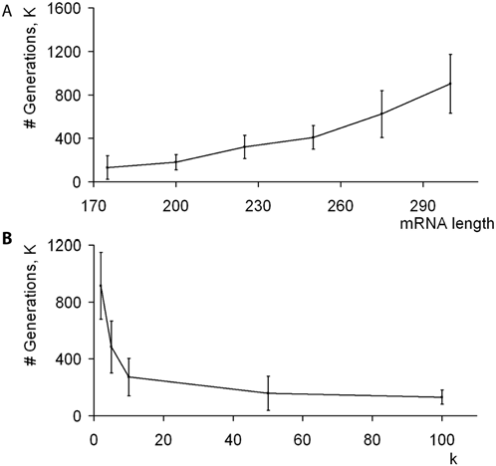
Effect on the speed of codon length evolution of (A) mRNA length, and (B) the parameter *k* (the contribution of coding system size to the replication function). The same parameter values were used in all simulations within each individual panel with the exception of the parameters corresponding to the *x* axis. Each data point on the graphs corresponds to the number of generations that is required for a population containing quadruplet tRNA rules to reach a state with equal number of quadruplet and triplet tRNA rules. Each data point represents the mean value for 10 simulations with different random starting conditions; error bars indicate the standard deviations of the distributions obtained.

The speed of codon size evolution depends on a number of simulation parameters. Among them, mRNA size and the coefficient responsible for the fitness of coding systems based on their size (k in Figure 1) are of a particular interest. Figure 3 shows the effect of these parameters on simulations of coding systems with initial quadruplet decoding. The plots in Figure 3 show the number of generations needed to achieve a state where the number of tRNA rules with triplet codons equals the number of tRNA rules with quadruplet codons.

The mRNA length correlates linearly with the time required to reach the state of triplet and quadruplet equality (Figure 3b) on the tested interval (note that that CPU-time required for such simulations increases more dramatically). This suggests that evolution of codon size is more rapid for coding systems with smaller proteomes. The speed of codon size evolution is increased by the parameter *k* defining the contribution of coding system size to the fitness function, which suggests that competition for energy and resources is a major contributing factor in compaction of the code. Therefore, if our model reflects the true history of the evolution of the genetic code, most transitions in codon sizes likely took place during the very early stages of protein world evolution.

## Discussion

**Figure 4:**
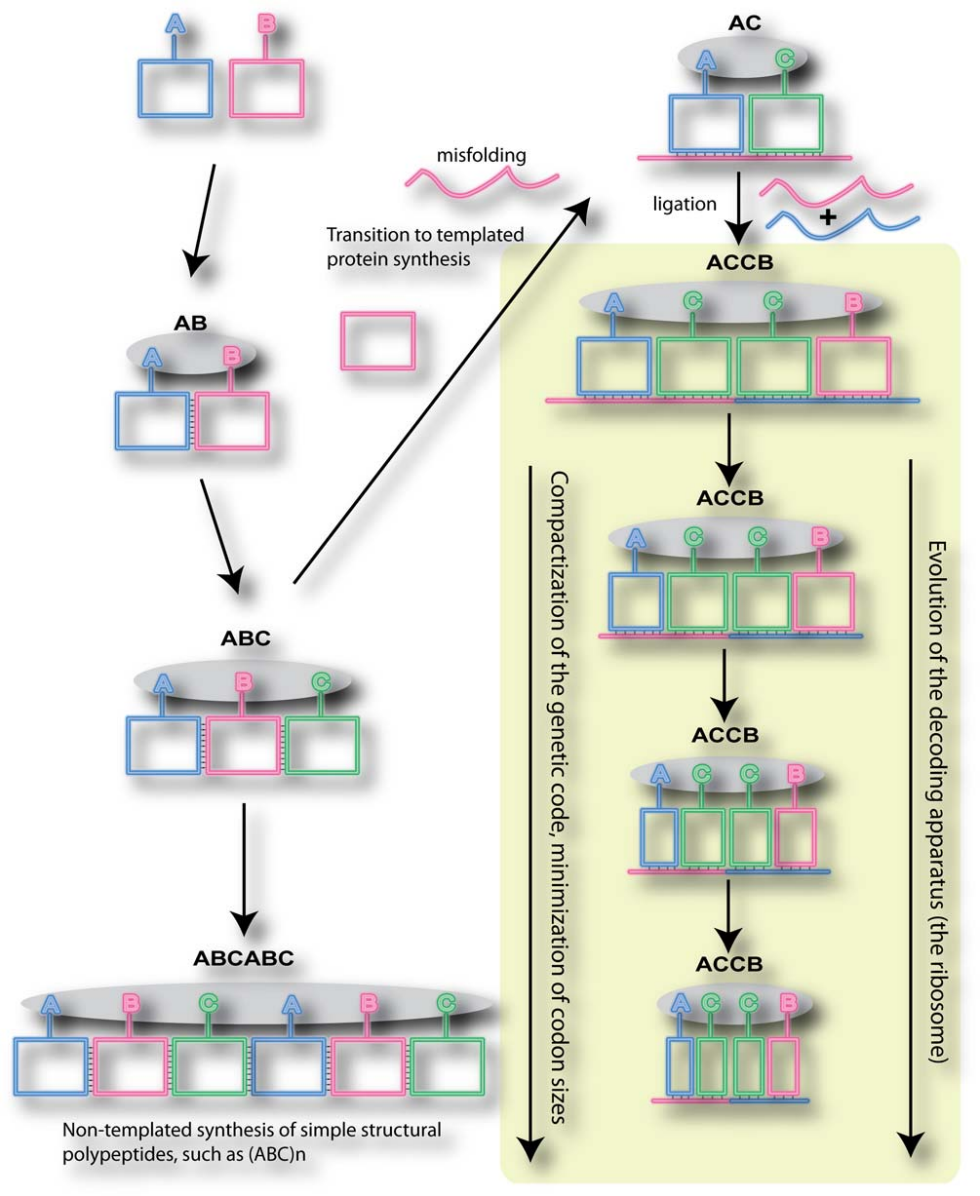
The codon size reduction hypothesis of the triplet genetic code origin. The model considers the possibility that initially dipeptide molecules in the RNA World were synthesized *via* peptidyl transfer between two interacting amino acid synthesizing ribozymes (shown as squares with attached letter symbols). Such encoding could be used to encode long polypeptides containing short amino acid repeats. A misfolded form of a ribozyme containing regions of complementarity to other ribozymes could be used as a template for the association of other ribozymes. Their combination and ligation would lead to the formation of longer templates capable of encoding a variety of complex protein molecules. The highlighted area of the scheme shows gradual reduction in the size of complementary interactions, a process modeled in the computer simulations described in this work.

The possibility of the evolution of triplet decoding from codes with codons of larger length is highly attractive, as it can explain how accurate protein biosynthesis could have been organized in the past without assistance from the modern decoding apparatus. A model of this is illustrated in Figure 4, and is conceptually similar to the one suggested recently [60]. It is reasonable to assume that the synthesis of amino acids preceded the synthesis of peptide products and that, in the absence of any proteins, the synthesis of amino acids was accomplished by ribozymes or their complexes [17]. Regardless of the role of individual amino acids in the RNA World, it is also logical to assume that formation of dipeptides preceded formation of longer peptides. Such an initial peptidyl-transferase reaction could be carried out by a simple prototype of the large ribosomal subunit, whose main component is RNA even in modern organisms [4], [6], [8]. Now consider two amino acids, A and B. If one particular dipeptide, say AB, carried properties that were more advantageous than of BA, it is likely that the ribozymes synthesizing amino acids A and B evolved to prefer a conformation favorable for AB-formation over BA-formation. The interactions stabilizing such a conformation could be based on simple complementary interactions between nucleotides of those ribozymes, as suggested by Orgel [61]. Addition of a third component into the system would allow encoding of ABC peptides as shown in Figure 4. By introducing similar complementary interactions between C and A synthesizing ribozymes, we create a system where non-templated coding of (ABC)_n_ polypeptides is possible. While it is unlikely that such a peptide would have any enzymatic properties, it may well have certain structural properties similar to fibrous proteins.

Taking into account that the RNA World was most likely populated by a variety of RNA molecules with a large spectrum of catalytic activities, and that nucleic acids are fragile and are unsuited for structural purposes, the first proteins (consisting of simple amino acid repeats) may have played structural roles as components of primordial membranes, or as matrixes for assembling cooperating RNA molecules. While the coding scheme shown in the left side of Figure 4 can be used to generate a variety of protein products consisting of simple amino acid repeats, it cannot be used for coding proteins with complex amino acid sequences, since this would require evolution of multiple aminoacyl-synthesizing ribozymes with a variety of complementary combinations between them, which in turn would abolish the possibility of accurate peptide biosynthesis. The solution can be provided by templated peptide synthesis, as illustrated in Figure 4. A misfolded ribozyme (B in Figure 4) could be used for the ordered association of two other ribozymes.

Further recombination/ligation events between such simple RNA molecules could generate templates encoding sufficiently complex protein products (primordial mRNAs). Such a system would not require a sophisticated decoding apparatus, since complementary interactions between ribozymes and templates are of sufficient length to provide stable and discriminative associations between them. Now, similar to the process that we modeled in this work *in silico*, we can envision evolution of such a system to shorten ribozyme-template interactions in parallel with evolution of the decoding apparatus (culminating in the modern ribosome) compensating for the loss of stability and discriminative power for such shortened interactions.

Such a hypothesis can be easily understood in terms of information coding theory [62]. One may consider the decoding apparatus as an evolving noisy channel through which information from RNA sequence is transmitted into protein sequence. The early decoding apparatus (or the lack of it) would have the information-processing properties of a low-quality transmitter with a high propensity for errors. This can be compensated by the length of the message itself in a manner similar to principles used in telecommunication, by adding redundancy to the message. As the transmission channel evolves, the redundancy can be partially removed to yield more compact and efficient code. Longer-than-triplet decoding allows a higher level of code degeneracy, and therefore makes the code more robust to the effect of mutations. For example, a genetic code based on quadruplets could be artificially designed so that a single substitution of a single nucleotide in nearly any position of the template would not affect the sequence of the encoded protein. Such decoding robustness may have been particularly beneficial in the primordial world, where replication was carried out with error-prone polymerases that have not evolved their accuracy to the levels of present-day enzymes.

## Methods

### Simulation initialization

The coding system χ_0_ is generated randomly within the limits defined by the parameters of a Codonevo simulation (see Codonevo program manual). At the first step, a set of tRNA rules τ_0_ is randomly generated for each of twenty different amino acids α. The number of tRNA rules for each amino acid (synonymous tRNA rules) is defined by the Codonevo parameters. The set of tRNA rules is not exhaustive, e.g. not all possible codons have associated tRNA rules. The sequence of mRNA μ_0_ is then generated from the codon parts of the tRNA rules, where mRNA length is parametrically defined in the beginning of a simulation.

At the second step, mRNA μ_0_ is translated using the set of tRNA rules τ_0_ to yield protein sequence π_0_, i.e., ξ:μ_0_×τ_0_→π_0_ (see subsection “Translation” describing the process of translation). Further, protein sequence π_0_ is used throughout the entire simulation as a reference sequence for estimating the fitness of protein sequences π_i_ generated by descendant coding systems. For simplicity, we consider evolution under purifying selection. Any deviation of protein sequence π_i_ from π_0_ in a descendant coding system penalizes its fitness, as measured by the protein fitness function φ, which is based on the alignment score of a protein π_i_ with protein π_0_ (see subsection “Protein fitness” describing computation of the function ϕ).

The number of offspring coding systems generated by each coding system in a population within a single generation is computed based on the replication function ρ (see subsection “Replication function”). Since the protein generated by the first coding system is the reference protein π_0_, its protein fitness function φ(π_0_) = 1 (see “Protein fitness” subsection below). While there is only one coding system in the population, coding system size does not contribute to the replication function; thus ρ = *m*, where *m* is a parameter of the Codonevo program (see Codonevo manual). The parameter *m* defines the maximum number of offspring coding systems produced by a coding system of average size within a given population.

### Birth-and-death cycles

Given the first generation, we continue the simulation using a birth/death process, for a number of generations (birth-and-death cycles). The maximum number of generations is defined by Codonevo parameters. The simulation process stops if it reaches the specified number of generations, or if the coding systems stop replicating, e.g. if the replication function ρ(χ_i_(T)) = 0 for every coding system χ_i_ in the population at generation T.

During the birth phase, the replication function ρ_i_ = ρ(χ_i_) is computed for each coding system χ_i_ of the population X(T), where T is the current generation and ρ_i_ is the number of coding system copies produced by χ_i_. For each χ_i_, a set of new coding systems Δ_i_ is generated, where Δ_i_ = {δ_i_^1^, …, δ_i_^s^}, δ_i_^j^ is an offspring coding system produced by χ_i_, and |Δ_i_| = ρ_i_. This yields a set of newly-born coding systems Δ(T) = {Δ_1_, …, Δ_n_}, where *n* = |X(T)|. At the end of a birth cycle, the entire population of coding systems consists of {X(T)∪Δ(T)}.

During the death phase, the subset Λ(T) of coding systems in {X(T)∪Δ(T)} are randomly destroyed until the total number of coding systems within this set reaches a certain limit O, i.e. so that |{X(T)∪Δ(T)}|−|Λ(T)|≤O. Then, the remaining set is assigned to be the set of coding systems in the population of the following generation:
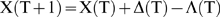
(1)


The parameter O represents environmental limitation of energy and food resources in a real world system and is defined as a parameter in the Codonevo program. Alternations of the birth and the death phases within each generation represent the cyclic nature of fluctuating rich/poor conditions in the real world, e.g. change of seasons, day and night alternations, etc. After a number of generations T_o_, the population size |X(T)| reaches the limit O, and|Δ(T)| = |Λ(T)| for any generation T>T_o_.

Coding systems in the set Δ are not precise copies of the coding systems in the set X. Instead, each copy in Δ is generated with small changes (mutations) in the composition or sequences of its elements (see subsection “Genetic variations” for a description of the mutation process).

### Replication function

We assume that the reproductive rate of a coding system positively correlates with the accuracy of the protein sequence produced by that coding system. In addition, large coding systems require more resources and time for reproduction.

Thus the rate ρ with which a coding system χ_i_ replicates depends on two components - the accuracy of the produced protein sequence, described by the protein fitness function φ(π_i_) (see “Protein fitness” subsection for details), and the deviation of the coding system's size s_i_ from the average coding system size in the population. The size *s_i_* of a coding system χ_i_ is defined as the sum of (1) the lengths of all μ_i_ and (2) all codons from the sets of tRNA rules τ_i_. The following equation is used to determine the number of offspring coding systems produced by a coding system χ_i_:
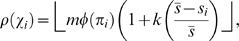
(2)where *m* and *k* are parameters of the Codonevo program. m can be defined as the maximum number of offspring coding systems produced by a coding system of a size equivalent to the mean size in a population. In practice, this limits the maximum number of offspring coding systems produced by coding systems of any size, as high deviations of size between coding systems within a population are very unlikely within the same generation. The parameter *k* defines the contribution of the effect of the size deviation to the replication function. When *k* = 0, no penalties are applied for deviations from the average size, whereas *k*>0 introduces a bonus or penalty for smaller or larger coding systems, respectively.

### Protein fitness

**Figure 5:**
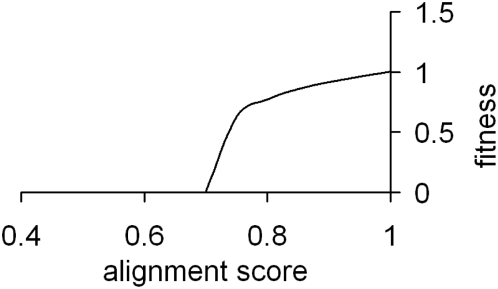
Plot of the pseudo-step function φ for calculating protein fitness. The function (see equation {3}) ensures infertility of coding systems producing protein sequences that are less than 70% identical to the reference sequence.

The protein fitness function φ for coding system χ_i_ is based on the score of the alignment of protein sequence π_i_ produced by coding system χ_i_ with the sequence of the reference protein π_0_. For simplicity, we model evolution under purifying selection, as most housekeeping proteins which encode genes evolve under strong purifying selection [63]. Hence, we consider any deviations of protein π_i_ from π_0_ to be deleterious, independent of the position where the changes between the two protein sequences occur. Therefore, the fitness function can be represented as an increasing function of a protein alignment score. It is reasonable to assume that a certain number of changes in a protein sequence should result in a complete loss of protein function and therefore in the coding system being unable to reproduce. We decided to set this limit to 0.7 (70% identity), where φ(π_0_) = 1, as this is a much higher level of similarity than what can be observed between distant homologs that retain the same functionality. We also assumed that a small number of sequence changes should affect the fitness function insignificantly. These considerations result in a behavior that is illustrated with the plot in the Figure 5. Using this curve as a target, we estimated the parameters of a protein fitness function φ that would yield the appropriate behavior; the resulting function is shown below.
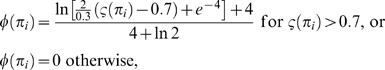
(3)where ζ is the alignment score (see “Alignment” subsection below).

### Alignment

Since we consider that all positions of a protein sequence are equally important, we perform position-unspecific alignment. As we do not model chemical properties of amino acids, any pair of amino acid substitutions is scored equally. Consequently, we simply assign a score of 7.5 for a match, −1 for a mismatch, −10 for opening a gap and −1 for elongation of a gap.
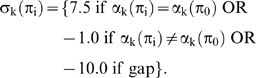
(4)


We define σ_k_(π_i_) as the score for the k^th^ position within an alignment of π_i_ and π_0_. A final score ζ(π_i_) of the alignment is computed as the sum of the above scores divided by the maximum possible score (i.e. for 100% identity):
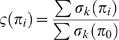
(5)


### Genetic variations

Point mutations take place in any nucleotide sequence of a digital coding system (its mRNA sequence and just the codon parts of tRNA rules). The number of mutations that happen in a new coding system is determined by a Gaussian distribution of which the standard deviation and the mean value are parameters defined by Codonevo parameters. The value is floored to have an integer value of mutations, and negative values are converted to zero. Particular types of mutations (substitutions, deletions, insertions) occur with respective probabilities of 0.5, 0.25 and 0.25. The location of a point mutation is determined randomly. Mutation rates remain constant throughout the entire simulation. If a deletion occurs in a codon of a tRNA rule consisting of only a single nucleotide codon, then the tRNA rule is removed entirely.

The second type of evolutionary event in Codonevo is gene duplication. In this case, a tRNA rule can be duplicated with a probability defined by a parameter of the Codonevo program (see Codonevo manual).

### mRNA translation

We model the process of translation using the tRNA rules τ to generate the protein (amino acid sequence) π from the mRNA μ. In this process, the tRNA rule with the best codon is chosen for the beginning of the mRNA sequence, and the corresponding amino acid is inserted into the beginning of the protein sequence π. In the following step, the process is repeated for the sequence of mRNA μ, starting from the point next to the previous codon-mRNA match. This process is continued iteratively for the entire mRNA.

To compute the best matching tRNA rule codon with the mRNA sequence, we use the following scoring system. For each codon pair between a tRNA rule codon and mRNA, matching nucleotides are given a score of 1.0, and mismatching nucleotides are given a score of −1.5. In addition, to make the process more natural, a bonus score is given to mimic stacking interactions between matching nucleotides, with each pair of adjacent matching nucleotides given an additional score 0.5. Then the amino acid α corresponding to the codon with the maximal matching score is inserted into the resulting protein sequence π. If more than one tRNA rule has the best matching score for the current mRNA location, a tRNA rule is chosen randomly from the pool of the best-scoring tRNAs.

It can be argued that our translation algorithm does not necessarily find the optimal sequence of the best matching tRNA rules across the entire mRNA. However, the step-wise approach in our algorithm reflects the natural course of translation, since during the natural process of translation, each tRNA is chosen based on a *local* mRNA sequence, independent of a sequence located downstream of the mRNA location currently being translated.

## Supporting Information

Movie S1 Dynamic histogram of codon and mRNA length evolution for the simulation corresponding to the bottom plot in Figure 1. The bar mRNA shows the sum of mRNA lengths from all coding systems in the evolving population. Other bars indicate a number of tRNA rules with particular codon lengths that are specified underneath each bar.
(0.07 MB MOV)
